# A narrative review of motor control dual pathways: the corticospinal and reticulospinal tracts in synergy and differentiation

**DOI:** 10.3389/fnana.2026.1768036

**Published:** 2026-04-10

**Authors:** Yun Xing, Jiaxin Liu, Linrui Wu, Ke Zhang, Shengbo Yang

**Affiliations:** Department of Anatomy, Zunyi Medical University, Zunyi, China

**Keywords:** axonal regeneration, corticoreticular pathway, corticospinal tract, motor control, neuromodulation, post-stroke recovery, reticulospinal tract, spasticity

## Abstract

The corticospinal tract (CST) and reticulospinal tract (RST) represent the core descending pathways within the central nervous system for motor control. This review elucidates the anatomical and functional interplay. Anatomically, the CST and RST fibers are spatially proximate within the spinal cord and are integrated into a continuous regulatory axis via the corticoreticular pathway. Functionally, they exhibit both synergy and specialization: the CST primarily governs contralateral distal limb fine motor control, whereas the RST, operating in a bilateral mode, regulates axial and proximal body movements, postural stability, and adaptation to strength training. Notably, following CST damage (e.g., stroke or spinal cord injury), the RST demonstrates remarkable plasticity and serves as a critical substrate for functional compensation and recovery. Furthermore, an imbalance in CST-RST function has been implicated in the pathophysiology of conditions such as post-stroke spasticity and multiple sclerosis. Therapeutic strategies targeting the CST-RST network, including neuromodulation and molecular interventions aimed at promoting axonal regeneration and modulating network excitability, present promising new directions for the treatment of neurological disorders. Future research should focus on deciphering the specific interactions at the spinal interneuron level to advance the development of precise rehabilitation strategies.

## 1 Introduction

Motor control is one of the most complex functions of the nervous system and relies on the coordinated interplay of multiple descending pathways for precise execution. Among these, the corticospinal tract (CST) and reticulospinal tract (RST) are two core descending pathways that have long been regarded as parallel systems mediating “fine motor control” and “postural control,” respectively (Soteropoulos et al., 2012). From an evolutionary perspective, the RST is an ancient and phylogenetically conserved pathway in vertebrates (Schading-Sassenhausen et al., 2025). In contrast, the CST has expanded markedly in mammals, particularly in primates, coinciding with the evolution of skilled motor abilities (Roze et al., 2025). This evolutionary sequence is recapitulated during ontogeny. During embryonic and early postnatal development, the RST develops and becomes functional before the CST, subserving early spontaneous movements (Forssberg, 1999). This developmental characteristic reinforces the classical neuroanatomical view that posits the CST is the dominant system of motor control. Its maturation and myelination are closely associated with the acquisition of fine motor skills, therefore establishing it as the “primary command pathway” for executing skilled, dexterous movements (Gutterman and Gordon, 2023). Conversely, the RST has been more strongly linked to relatively primitive motor functions, such as postural maintenance, balance, and orientation (Baker, 2011). Furthermore, while both tracts receive sensory feedback, they process it differently. The CST processes fine tactile and proprioceptive information for movement adjustment (Kalambogias and Yoshida, 2021), whereas the RST likely integrates vestibular and gross whole body posture signals to maintain balance (Takakusaki, 2017).

However, the advent of advanced neural tracing, electrophysiological techniques, and neuroimaging has challenged this simple functional dichotomy. A growing body of evidence has revealed that the CST and RST exhibit far more intricate anatomical and functional interactions than previously conceived. Anatomically, they do not descend in isolation. The cerebral cortex forms a cascading connection with the RST via the corticoreticular pathway, constituting a continuous functional axis from higher-order motor cortices to spinal motor neurons (Fisher et al., 2012). At the spinal level, the terminal arborizations of the CST and RST fibers are not only spatially proximate but also functionally integrated, likely through shared spinal interneuronal networks, to coordinate the excitation and inhibition of bilateral muscle groups (Riddle and Baker, 2010). At the systemic level, both tracts are embedded within the broader cortical–basal ganglia–thalamic–brainstem and cerebellar circuits, which collectively support higher-order motor planning and coordination (Côté et al., 2018; Arber and Costa, 2022). These findings compel a re-evaluation of their relationship in motor control: they are not merely “principal and supporting actors” but rather constitute parallel processing systems that are both functionally synergistic and specialized.

This functional synergy and specialization become particularly evident in the pathogenesis and recovery processes of neurological disorders. Following damage to these motor pathways, such as stroke or spinal cord injury, the RST demonstrates remarkable plasticity. Functional compensation is a critical neural substrate for motor recovery (Baker et al., 2015). Therefore, a deeper understanding of the interactive mechanisms between the CST and RST is of significant theoretical importance. Furthermore, it is clinically imperative to elucidate the pathophysiology of neurological disorders (e.g., post-stroke spasticity and dystonia) and to develop novel therapeutic strategies based on neuromodulation and molecular targeting.

Based on this rationale, this review aimed to provide a narrative synthesis of the anatomical foundations and fiber connectivity of the CST and RST, along with their functional synergies and specializations across multiple levels. It subsequently elaborates on the pathological alterations and compensatory mechanisms in common neurological disorders. Finally, we explore emerging therapeutic strategies targeting the CST-RST network to provide novel perspectives on both the fundamental understanding of motor control mechanisms and clinical rehabilitation practices.

## 2 Anatomical architecture and localization techniques for the CST and RST

### 2.1 Anatomical and functional characteristics of the CST

In humans, the CST originates primarily from the primary motor cortex (M1), with additional significant contributions from the premotor cortex, supplementary motor area (SMA), and primary somatosensory cortex (Seo and Jang, 2013; Chenot et al., 2019). Additional fibers also arise from the pre-supplementary motor area, Brodmann area 5, the caudal cingulate zone, and the posterior part of the rostral cingulate zone (Usuda et al., 2022). The fibers descend through the internal capsule, cerebral peduncles, and basis pontis to reach the medullary pyramid. During pyramidal decussation, the majority (approximately 75–90%) of the fibers cross the midline to form the lateral CST within the contralateral spinal cord lateral funiculus, which primarily innervates the limb muscles. A smaller uncrossed portion continues ipsilaterally as the anterior CST within the ventral funiculus. Most fibers of this anterior tract ultimately decussate at segmental spinal levels, working in concert with a minor persistent ipsilateral component to supply the trunk muscles (Usuda et al., 2025). This anatomical organization underlies the bilateral cortical control of the trunk muscles, in contrast to the predominantly contralateral control of the limb muscles. In rodents, the CST primarily modulates movement through interneurons in laminae IV–VIII of the spinal cord. In primates, however, the CST is biased toward direct motor execution. Approximately 80.5% of corticospinal projections form monosynaptic connections with anterior horn alpha motor neurons to finely control distal muscles, while the remaining 19.5% terminate on spinal interneurons (Sinopoulou et al., 2022).

### 2.2 Anatomical and functional connectivity of the RST

In vertebrates, the RST originates predominantly from the medial pontomedullary reticular formation, with its core nuclei including the oral and caudal pontine reticular nuclei and the gigantocellular nucleus in the medulla (Matsuyama et al., 2004). Axons from these nuclei descend primarily within the ipsilateral anterior funiculus and the anteromedial portion of the lateral funiculus of the spinal cord, terminating in laminae VII and VIII. A significant proportion of these fibers decussate, constituting the anatomical basis of their bilateral innervation pattern (Liang et al., 2015). The RST influences motor neurons through both the monosynaptic and disynaptic pathways, and its principal functional output is critically dependent on integration within spinal interneurons (Riddle et al., 2009). Furthermore, this pathway projects to the spinal dorsal horn, indicating a direct modulatory role in sensory processing (Heinricher et al., 2009).

### 2.3 Localization techniques for the CST and RST

Precise localization of the CST and RST relies on multiple neural tracing and neuroimaging techniques. For the CST, detailed anatomical mapping can be achieved through enzyme histochemical methods such as protein kinase C gamma immunolabeling (Zhang et al., 2019), while its projection trajectories and terminal arborization are resolved using anterograde tracers, including biotinylated dextran amine (Ferguson et al., 2001). Manganese-Enhanced Magnetic Resonance Imaging enables *in vivo* dynamic tracking, with results validated against noninvasive techniques, including Diffusion tensor imaging (Lin et al., 2001). For the RST in primates, its originating nuclei are specifically labeled using retrograde tracers such as Wheat Germ Agglutinin-conjugated Horseradish Peroxidase, adenoviral vectors, and Fluorogold (Sakai et al., 2009), while its descending projections are delineated via anterograde tracing techniques employing biotinylated dextran amine (Fregosi et al., 2017). Emerging methodologies, including retrograde viral tracing combined with tissue clearing and three-dimensional imaging, provide powerful tools for resolving the distinct spatial trajectories of both tracts (Inoue and Ueno, 2025).

## 3 Functional connectivity of CST and RST fiber trajectories

### 3.1 Spatial proximity and parallel pathways of CST and RST

Within the spinal cord, RST fibers exhibit significant spatial proximity to the CST, descending closely along the anterior border of the lateral CST (Nathan et al., 1996). Anatomical adjacency provides a structural foundation for functional interactions. Evolutionary studies have indicated that in primates, the reticulospinal system may constitute a pathway parallel to the CST for controlling distal muscles, thereby enhancing the complexity of motor control (Riddle et al., 2009). The neuronal population in humans that mediates ipsilateral motor evoked potentials is distinct in its cortical origin from the population that activates the fast-conducting CST, thereby providing physiological evidence for the RST as a parallel descending pathway independent of the CST (Chen et al., 2003).

### 3.2 Pathway integration at supraspinal levels

**Figure 1:**
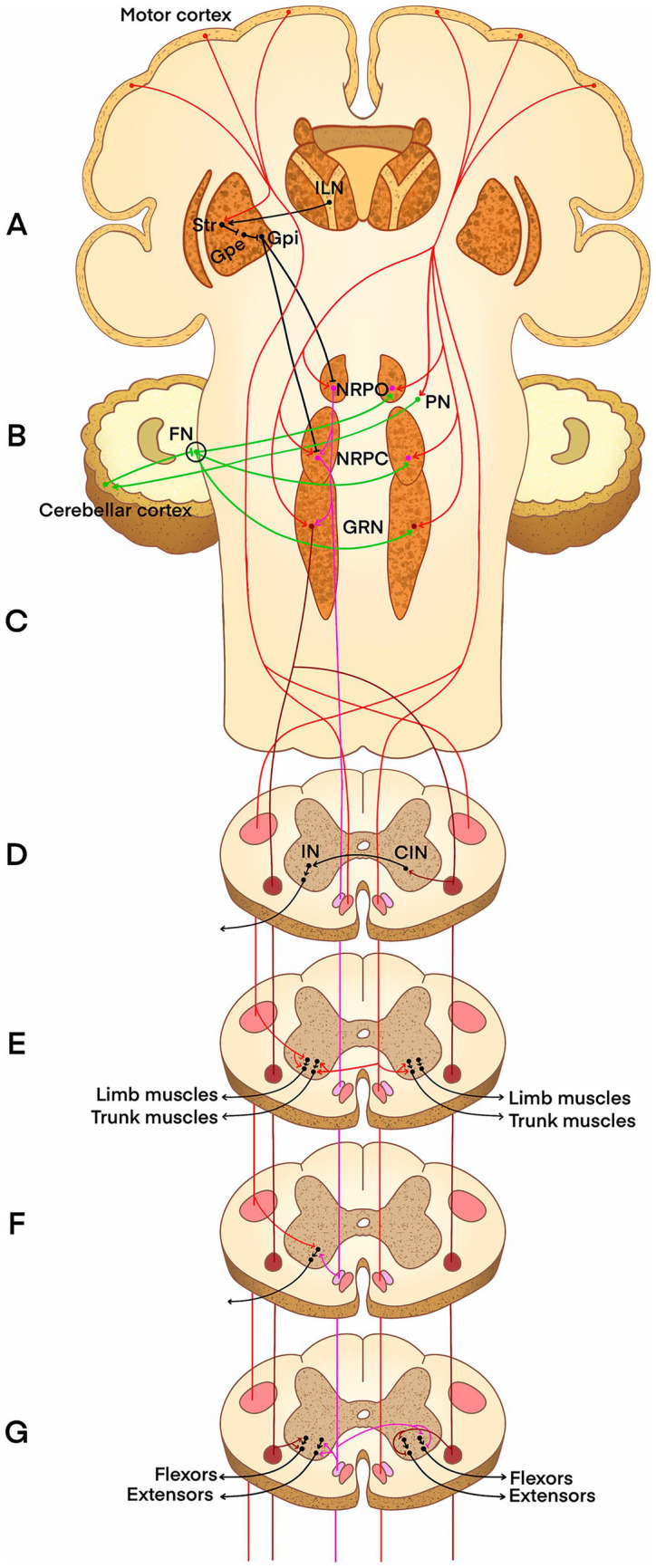
Illustration of the synergy and differentiation between the CST and RST. **(A)** The striatum (Str) integrates excitatory inputs from the cerebral cortex and the intralaminar nuclei of the thalamus (ILN) and outputs inhibitory signals to the globus pallidus externus (GPe). Then, the GPe sends inhibitory signals to the globus pallidus internus (GPi), which projects to the nucleus reticularis pontis oralis (NRPO) and caudalis (NRPC). **(B)** The cerebral cortex regulates the contralateral cerebellum via the pontine nuclei (PN). Subsequently, the inhibitory output from cerebellar Purkinje cells modulates the pontine and medullary reticular nuclei primarily through the fastigial nucleus (FN). **(C)** At the brainstem level, most fibers of the corticospinal tract (CST) decussate to the contralateral side, and some fibers of the reticulospinal tract (RST) also decussate to the contralateral side. **(D)** Activation of the contralateral RST by the CST excites the spinal commissural interneurons (CINs), which ultimately modulate the ipsilateral ventral horn interneurons (INs). **(E)** Within the CST, the lateral CST innervates limb muscles, while the anterior CST innervates bilateral trunk muscles. **(F)** A proposed hypothesis that the CST and RST may share a common IN. **(G)** The medial RST descends in the anterior funiculus, facilitating ipsilateral extensors. It gives off a subset of fibers that cross via the anterior white commissure to inhibit contralateral flexors. The lateral RST facilitates ipsilateral flexors and inhibits contralateral extensors. In the figure, bright red lines (CST), pink lines (medial RST), and burgundy lines (lateral RST) denote the spinal tracts, with the circles in the spinal cord cross-section indicating their relative positions. The black lines in the brain represent supraspinal integration, while those at the spinal level represent fibers originating from interneurons and motor neurons. The green lines represent cerebellar regulation. Symbols “↓” and “⊥” indicate excitatory and inhibitory synapses.

At the cortical and diencephalic levels, the CST and RST are co-embedded within cortical–basal ganglia–thalamic–brainstem circuits. Specifically, the striatum integrates excitatory inputs from the cerebral cortex and the intralaminar nuclei of the thalamus, and subsequently projects inhibitory outputs to the external segment of the globus pallidus externus (Kang et al., 2021). In turn, the globus pallidus externus sends inhibitory projections to the internal segment of the globus pallidus internus (Groenewegen, 2003), which provides inhibitory signals to the ipsilateral oral and caudal pontine reticular nuclei (Figure 1A, pathway in black) (Mena-Segovia and Bolam, 2017). This circuit exerts a modulatory influence over descending motor signals at this level, thereby participating in motor initiation, selection, and regulation (Kang et al., 2021).

### 3.3 Cerebellum serves as a regulatory nexus

The cerebellum represents another critical node of dynamic interactions between the CST and RST. Motor commands from the cerebral cortex are relayed via the ipsilateral pontine nuclei and predominantly project to the contralateral cerebellar hemisphere (Wu et al., 2023). Subsequently, the Purkinje cells of this cerebellar hemisphere send inhibitory signals to modulate its deep cerebellar nuclei, primarily the fastigial nucleus, thereby ultimately attenuating the descending excitatory brainstem commands that are ipsilateral to the original cerebral cortex. These commands originate from the oral and caudal pontine reticular nuclei, as well as the gigantocellular reticular nucleus (Figure 1B, pathway in green) (Takahashi and Shinoda, 2021).

### 3.4 Pivotal role of spinal interneurons in CST and RST interactions

Spinal interneurons serve as critical hubs for the functional convergence and divergence of the CST and RST. In addition to activating the ipsilateral RST (Cleland and Madhavan, 2021), the CST can also influence motor output by engaging the RST that has decussated to the contralateral side. At the brainstem level, a contingent of fibers within the RST decussates to the contralateral side (Figure 1C, pathway in burgundy), exciting commissural interneurons in the contralateral spinal cord. The excitation of these commissural interneurons facilitates ipsilateral premotor interneurons within the ventral horn to regulate ipsilateral movement (Figure 1D, pathway in burgundy and black). This trans-spinal pathway is essential for coordinating bilateral movements, such as locomotion (Edgley et al., 2004). In humans and certain primates, the CST predominantly forms direct monosynaptic connections with motor neurons to control fine distal movements of the limbs, while also engaging in synaptic contacts with spinal interneurons. In cats and rodents, the CST primarily integrates information for proximal limb and trunk movements through indirect, interneuron-mediated pathways (Figure 1E, pathway in bright red) (Williams et al., 2017). The RST establishes dense and extensive axonal contacts with spinal interneurons, exerting its modulatory effects (Mitchell et al., 2016). Electrophysiological evidence has indicated that CST activation can facilitate RST-mediated control of proximal muscles, and that both tracts may share interneurons to influence motor execution, highlighting their functional synergy at the spinal level (Figure 1F, pathway in pink and bright red) (Li et al., 2024).

### 3.5 Core integrative pathway: the corticoreticulospinal axis

Functional integration between the CST and RST is achieved via the corticoreticular pathway, forming a continuous control axis from the cortex to the spinal cord (Taga et al., 2021). This pathway originates in the motor cortex, projects to the pontomedullary reticular formation, and subsequently descends to the spinal cord via the reticulospinal neurons, ultimately influencing motor neurons through either interneurons or direct connections (Fisher et al., 2012). Phylogenetic analyses revealed that while the role of the CST became more prominent during evolution (e.g., forming direct corticomotoneuronal connections in primates), the corticoreticular pathway, although relatively diminished, retained plasticity and played a critical compensatory role after CST injury (Alstermark and Pettersson, 2014). Furthermore, the corticoreticulospinal projection system is inherently more amenable to regeneration than the CST system, highlighting the evolutionary principles of motor control and its potential for recovery (Asboth et al., 2018). In addition, while the cerebral cortex exerts direct control over the spinal cord via the CST (Liu et al., 2021), it indirectly modulates the RST through corticoreticular projections, thereby transforming high-level motor intentions into specific executive commands (Matsuyama et al., 2004).

## 4 Functional synergy and specialization of the CST and RST

### 4.1 Division of labor and collaboration in motor control

The CST and RST exhibit clear functional differentiation in motor control. The CST is primarily responsible for the fine motor control of the contralateral distal limbs, such as independent finger movements and manipulation. In contrast, the RST primarily regulates the trunk and proximal muscles, governing postural stability, gross motor movements, and whole-body anticipatory postural adjustments (Viseux et al., 2025). However, this division of labor is not absolute; the RST can also influence hand muscles via monosynaptic and disynaptic connections and serves as a critical substrate for functional compensation following CST damage (Baker, 2011). Furthermore, the fundamental patterns of muscle innervation differ. The CST predominantly exerts contralateral control, primarily facilitating the activity of the contralateral extensor and flexor muscles (Cheney et al., 1991). In contrast, the RST exhibits a complex pattern of bilateral control: the medial RST (of pontine origin) facilitates the ipsilateral extensors and inhibits the contralateral flexors, whereas the lateral RST (of medullary origin) facilitates the ipsilateral flexors and inhibits the contralateral extensors (Figure 1G, pathway in pink and burgundy) (Zonnino et al., 2021). Overall, the RST demonstrates a conserved tendency to facilitate the ipsilateral flexors and contralateral extensors, a pattern conserved across multiple species (Davidson et al., 2007). Furthermore, transcranial magnetic stimulation (TMS) studies have demonstrated that while the CST plays a dominant role in force output during young adulthood, the RST’s contribution to force maintenance increases significantly with aging. This shift reflects a dynamic functional reorganization between these two descending pathways during the aging process (Maitland and Baker, 2021).

### 4.2 Neural adaptations in strength training

The RST plays a predominant role in the neural adaptations induced by strength training. Emerging evidence indicates that synaptic remodeling associated with strength training occurs primarily within the intracortical circuits of M1 and the reticulospinal system rather than within the CST or motor neurons themselves. This highlights the pivotal role and therapeutic potential of the corticoreticulospinal system in mediating strength gains and facilitating motor recovery after neurological injury (Glover and Baker, 2020).

### 4.3 Common pathways for mitigating muscle overactivity

Both the CST and RST can contribute to mitigating muscle overactivity, albeit through distinct mechanisms. A reduction in output from the CST originating in M1 can directly lead to a decrease in muscle force generation (Angius et al., 2016). In contrast, the SMA, via its robust corticoreticular projections, can enhance descending drive through the lateral RST. This increased drive may activate inhibitory interneurons within spinal reciprocal inhibitory circuits, thereby suppressing muscle overactivity and improving movement coordination (Hirabayashi et al., 2020). This suggests that both tracts can achieve functional synergy in motor control by modulating spinal inhibitory networks.

## 5 Alterations in the CST and RST following brain and spinal cord injury

### 5.1 Stroke

Impairment and subsequent recovery of motor function following stroke are primarily determined by the extent of damage to the CST and integrity of alternative pathways, such as the corticoreticular pathway (Liu et al., 2021). In a rodent model of unilateral motor cortex stroke, the contralesional, intact cortex indirectly modulates denervated cervical spinal circuits by enhancing its connectivity with the medullary reticular formation, thereby facilitating functional recovery. This confirms that post-stroke motor function is dependent upon the intact cortex and its indirect corticoreticular pathway (Bachmann et al., 2014). Investigations in primates have demonstrated that recovery of upper limb function following extensive sensorimotor cortex lesions is closely associated with a significant upregulation of corticoreticular projections originating from the intact SMA and terminating in the gigantocellular nucleus of the medulla. This finding suggests that this pathway can compensate for motor control of the upper limb when corticospinal projections are compromised (Darling et al., 2018). The development of post-stroke spasticity is closely associated with an imbalance in descending inhibition, particularly involving the RST and vestibulospinal tract (Miller et al., 2014). Following CST damage, the RST undergoes adaptive changes that enhance its influence on spinal interneurons and motor neurons, thereby constituting a critical substrate for functional compensation (Fisher et al., 2012). Specifically, the RST may compensate for the loss of CST input, providing some proximal recovery of isolated and within-synergy movements, but deficits in performing out-of-synergy movements and finger fractionation remain (Mooney et al., 2024). The role of the RST in gross motor function is attributed to its capacity to connect large muscle groups in a synergistic manner, whereas the CST innervates smaller motoneuron pools specialized for fine motor control. Increased excitability of the RST has been confirmed to be closely associated with motor recovery, force output, and the development of spasticity after stroke, which is largely enabled by its extensive collateral projections (Akalu et al., 2023). In the early stages of rehabilitation following internal capsule hemorrhage, functional recovery is primarily mediated by the cortico-rubral tract. However, when this pathway is compromised, the corticoreticular pathway can be dynamically recruited to assume a compensatory role, revealing a mechanism of functional reorganization among cortico-brainstem pathways (Ishida et al., 2019). Recent research suggests that normative atlases of the CST and corticoreticular pathway, generated using methods such as direct streamline normalization, may facilitate more precise calculation of regional damage and plasticity in stroke studies (Boyne et al., 2025).

### 5.2 Brainstem infarction

Brainstem infarctions can directly affect the origins or fibers of both the CST and RST. In acute brainstem infarction, the integrity of the CST exhibits dynamic changes across different stages of the disease process, and sterile inflammation persists throughout, influencing the secondary damage process of the CST (Shi et al., 2024). Longitudinal neuroimaging follow-up confirmed that the damaged CST retains a degree of reversibility even after prolonged ischemia, providing evidence for an extended therapeutic time window (Li et al., 2018). Techniques such as diffusion tensor imaging can be utilized to assess the extent of CST damage, predict functional prognosis, and visualize the reorganization process (Cho et al., 2007). Primate studies have revealed that following damage to primary motor areas, corticoreticular projections from the SMA to the brainstem reticular formation undergo compensatory upregulation, thereby driving functional recovery (Darling et al., 2018). Furthermore, a case report of right-sided pontine infarction has demonstrated that injury to the CST within the pontine base results in delayed recovery of fine motor control in the contralateral limbs. In contrast, the RST, located in the tegmentum, remains largely preserved due to its bilateral corticoreticular inputs, enabling patients to achieve rapid recovery of gross motor functions, such as postural control, in the early stages. This clinical observation underscores the core role and therapeutic potential of the corticoreticulospinal axis in motor compensation (Lei, 2024).

### 5.3 Parkinson’s disease

Compensatory mechanisms in Parkinson’s disease (PD) may explain the temporal delay between the onset of neurodegenerative processes and the emergence of clinical symptoms. The structural integrity of the CST is believed to contribute to this compensation in PD. Diffusion tensor imaging analysis reveals that PD patients exhibit reduced fractional anisotropy values in the CST, and this tract undergoes progressive changes correlating with disease advancement (Dayan et al., 2024). PD exerts a direct influence on M1 and its output pathways, an effect that originates during the premotor phase, with structural changes emerging in the early stage of the disease (Fu et al., 2022). The pathophysiology of PD involves not only the CST but also impairment within the RST, contributing to postural instability and gait disturbance (Pötter-Nerger et al., 2008; Takakusaki et al., 2023). Disruption of brainstem circuitry, including the gigantocellular nucleus, in PD compromises gait automaticity, necessitating increased reliance on cognitive resources for locomotor control (Farhani et al., 2026). Behavioral studies and deep brain stimulation interventions have demonstrated that PD patients with pronounced postural instability and gait disturbance exhibit impairments in rapid movement initiation mediated by the RST (Thevathasan et al., 2011). The RST plays a critical role in anticipatory postural adjustments by modulating inhibitory interneurons (Silva-Batista et al., 2024).

### 5.4 Spinal cord injury

In spinal cord injury, both the CST and RST are frequently compromised. In cervical spondylotic myelopathy, clinical symptoms are significantly correlated with volume loss affecting the lateral CST, rubrospinal tract, and ventral-ventrolateral RST (Hopkins et al., 2018). In human clinical studies, it has been observed that following CST injury, the RST participates in compensatory motor control through mechanisms such as axonal sprouting (Sangari and Perez, 2020). Studies in non-human primates have demonstrated that after cervical CST lesions, motor recovery relies on dynamic cortical reorganization, characterized by a shift from early bilateral M1 activation to later extensive recruitment of contralateral M1 and premotor cortices (Nishimura et al., 2007). Recovery of motor function after unilateral pyramidal tract injury primarily relies on enhanced synaptic excitatory input from the RST to motor neurons innervating forearm flexors and intrinsic hand muscles; however, this compensatory mechanism exhibits a flexor bias (Zaaimi et al., 2012). TMS studies have confirmed that the RST possesses functional plasticity and is subject to selective modulation by contralateral motor commands (Tazoe and Perez, 2014). Following the CST injury in humans, the RST can undergo functional compensation, thereby contributing to the maintenance of proximal motor function (Liu et al., 2025). Following severe spinal cord contusion, neuromodulation of lumbosacral circuits enables the motor cortex to re-establish adaptive control over paralyzed hindlimbs via the residual glutamatergic RST (Asboth et al., 2018). Rodent research has indicated that the ventrolateral funiculus, which contains RST fibers, is crucial for movement initiation, and its integrity is a prerequisite for eliciting volitional movement (Cao et al., 2005). Furthermore, demyelinating diseases such as multiple sclerosis often selectively damage the lateral CST and RST. This leads to a reduction in the inhibitory input, resulting in a relative predominance of excitatory drive from the medial RST and vestibulospinal tract, which ultimately manifests as spasticity (Brown, 1994). Monitoring CST damage and RST preservation via magnetic resonance imaging may serve as an early biomarker for predicting the development of spasticity and mobility outcomes, which is crucial for continuous monitoring and rehabilitation planning (Schading-Sassenhausen et al., 2025). Notably, the medial fibers of the lateral RST are often partially spared because of their anatomical location, making perilesional reorganization a potential target for promoting functional recovery (Cao et al., 2005).

## 6 Treatment strategy

### 6.1 Neuromodulation techniques

TMS is an effective modality for concurrently targeting both the CST and RST. Stimulation of M1 with TMS not only directly activates the CST but also transsynaptically modulates the excitability of the brainstem reticular formation through corticoreticular projections, thereby influencing RST excitability (Russell et al., 2022). Experimental evidence indicates that the ipsilateral muscle responses evoked by high-intensity TMS over M1 are posture dependent, suggesting that this effect is primarily mediated by bilateral brainstem pathways, including the RST, rather than being transmitted directly through the CST (Fisher et al., 2012).

### 6.2 Molecularly targeted therapies

Studies in rodents have shown that at the molecular level, the promotion of CST axonal regeneration is a core strategy for motor function repair. Key transcription factors such as KLF6 have been shown to significantly enhance CST regeneration and functional restoration after spinal cord injury when coexpressed with Nr5a2 (Venkatesh et al., 2021). Upregulation of PI3Kδ has been shown to enhance axonal regeneration and functional recovery of the CST following spinal cord injury (Karova et al., 2025). Furthermore, it has been noted that molecular targets for CST axonal regeneration are primarily centered around neuronal subpopulation-specific genes, including Epha4, Efna5, Epha6, Epha7, Crim1, and Klhl14. At present, these molecules have only been identified as candidate genes associated with target selection for projections, and their specific functions in reorganization following injury remain to be elucidated (Inoue and Ueno, 2025). Concurrently, investigations into the molecular characteristics of neurons of origin for both the CST and the RST have identified novel targets for precision intervention. Recent single-cell transcriptomic analyses have revealed significant spatial and functional heterogeneity in the gene expression profiles of CST neurons. These specific molecular signatures determine their axonal projection targets and regenerative capacity (Golan et al., 2023). Within the reticulospinal system, researchers have also identified specific transcription factors, such as Lhx3 and Chx10, which serve as molecular markers defining glutamatergic neuronal subpopulations in the brainstem responsible for executing distinct motor commands (Bretzner and Brownstone, 2013). Of particular significance, a specific population of glutamatergic neurons located in the ventral medullary reticular formation in rodents has been demonstrated to selectively modulate fine motor control of the forelimb, such as reaching and grasping movements. This finding suggests that the RST is not solely involved in gross motor control but also encompasses discrete neural circuits dedicated to skilled motor tasks (Esposito et al., 2014). The discovery of these specific molecular markers and functional subpopulations lays a robust theoretical foundation for the future development of gene therapies or optogenetic approaches targeting discrete neural circuits, thereby enabling selective modulation of CST or RST function to promote motor recovery (Ruder et al., 2021).

## 7 Summary and future perspectives

This review elucidated the interactive roles of the CST and RST in motor control. Anatomically, these tracts are integrated into a functional axis via the corticoreticular pathway and exhibit significant spatial proximity at the spinal level. Functionally, they demonstrate a clear division of labor and synergy; the CST predominantly governs fine motor control of the contralateral distal limbs, whereas the RST is responsible for postural control and gross motor functions. This synergistic interaction is particularly evident in contexts such as strength training and the mitigation of muscle overactivity. Notably, the RST demonstrates remarkable plasticity following CST injury, establishing itself as a critical substrate for functional compensation. This finding holds significant clinical value for rehabilitation in stroke and spinal cord injury. Therapeutic strategies targeting the CST-RST network, including neuromodulation and molecular interventions, show considerable promise. Regarding the fine anatomical morphology and plasticity mechanisms discussed herein, the relevant conclusions are drawn primarily from animal studies. Although animals share a high degree of homology with humans in motor system development, it must be recognized that the unique postural control demands of human bipedalism and the complexity of post-injury recovery cannot be fully recapitulated in animal models. Future research should prioritize the following directions: employing emerging neural tracing technologies to elucidate the specific connectivity mechanisms between the CST and RST at the level of spinal interneurons, developing targeted rehabilitation strategies capable of selectively modulating the RST pathway, and investigating the distinct functional contributions of molecularly defined neuronal subpopulations to motor control. A deeper investigation of these questions will advance the theoretical framework of motor control and provide novel perspectives for the precise rehabilitation of patients with neurological disorders.
